# Impact of Age-Associated Cyclopurine Lesions on DNA Repair Helicases

**DOI:** 10.1371/journal.pone.0113293

**Published:** 2014-11-19

**Authors:** Irfan Khan, Avvaru N. Suhasini, Taraswi Banerjee, Joshua A. Sommers, Daniel L. Kaplan, Jochen Kuper, Caroline Kisker, Robert M. Brosh

**Affiliations:** 1 Laboratory of Molecular Gerontology, National Institute on Aging, National Institutes of Health, NIH Biomedical Research Center, Baltimore, Maryland, United States of America; 2 Department of Biomedical Sciences, Florida State University College of Medicine, Tallahassee, Florida, United States of America; 3 Rudolf Virchow Center for Experimental Biomedicine, Institute for Structural Biology, University of Würzburg, Würzburg, Germany; Saint Louis University, United States of America

## Abstract

8,5′ cyclopurine deoxynucleosides (cPu) are locally distorting DNA base lesions corrected by nucleotide excision repair (NER) and proposed to play a role in neurodegeneration prevalent in genetically defined Xeroderma pigmentosum (XP) patients. In the current study, purified recombinant helicases from different classifications based on sequence homology were examined for their ability to unwind partial duplex DNA substrates harboring a single site-specific cPu adduct. Superfamily (SF) 2 RecQ helicases (RECQ1, BLM, WRN, RecQ) were inhibited by cPu in the helicase translocating strand, whereas helicases from SF1 (UvrD) and SF4 (DnaB) tolerated cPu in either strand. SF2 Fe-S helicases (FANCJ, DDX11 (ChlR1), DinG, XPD) displayed marked differences in their ability to unwind the cPu DNA substrates. Archaeal *Thermoplasma acidophilum* XPD (taXPD), homologue to the human XPD helicase involved in NER DNA damage verification, was impeded by cPu in the non-translocating strand, while FANCJ was uniquely inhibited by the cPu in the translocating strand. Sequestration experiments demonstrated that FANCJ became trapped by the translocating strand cPu whereas RECQ1 was not, suggesting the two SF2 helicases interact with the cPu lesion by distinct mechanisms despite strand-specific inhibition for both. Using a protein trap to simulate single-turnover conditions, the rate of FANCJ or RECQ1 helicase activity was reduced 10-fold and 4.5-fold, respectively, by cPu in the translocating strand. In contrast, single-turnover rates of DNA unwinding by DDX11 and UvrD helicases were only modestly affected by the cPu lesion in the translocating strand. The marked difference in effect of the translocating strand cPu on rate of DNA unwinding between DDX11 and FANCJ helicase suggests the two Fe-S cluster helicases unwind damaged DNA by distinct mechanisms. The apparent complexity of helicase encounters with an unusual form of oxidative damage is likely to have important consequences in the cellular response to DNA damage and DNA repair.

## Introduction

Oxidative DNA damage represented by a spectrum of bases or sugar modifications is incurred by reactive oxygen species that arise from endogenous biochemical processes and can also be induced exogenously by environmental agents such as chemical compounds (e.g., aldehydes, peroxides) or ionizing radiation. Oxidative DNA lesions in nuclear and/or mitochondrial genomes result in perturbations to cellular DNA replication and transcription; furthermore, their accumulation predisposes individuals to accelerated tissue aging, neurodegeneration, and cancer. A variety of oxidative DNA lesions exist, and recent efforts have focused on establishing meaningful relationships between the accumulation of a particular oxidative lesion and aberrant cellular and organismal phenotypes as well as the pathways for repairing and tolerating the spectrum of oxidative DNA lesions [1].

**Figure 1:**
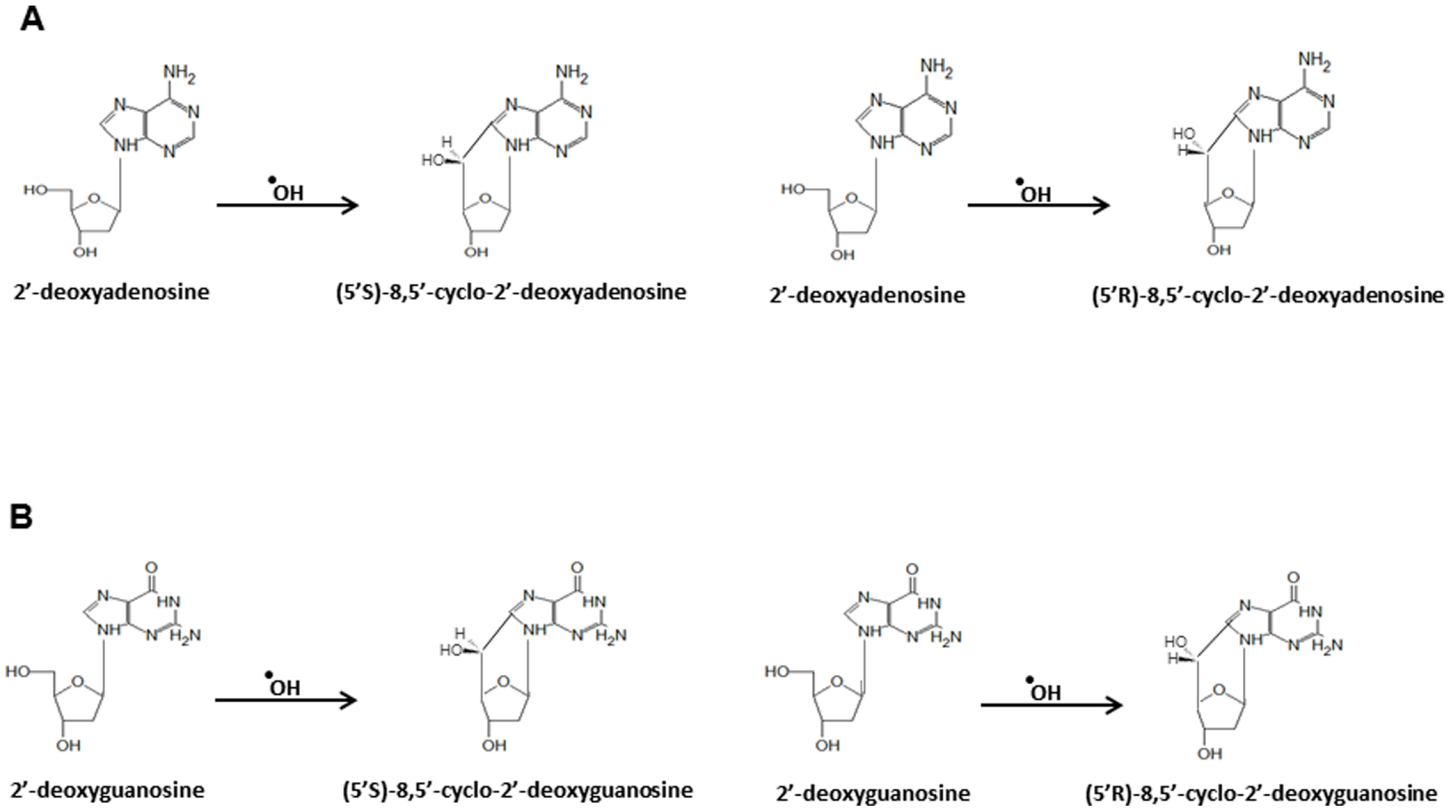
Conversion of adenine and guanine bases to cyclo dA and cyclo dG bases. *A,* Formation of 2′-deoxyadenosine to (5′S) 8,5′-cyclo-2′-dA and (5′R) 8,5′-cyclo-2′-dA. *B,* Formation of 2′-deoxyguanosine to (5′S) 8,5′-cyclo-2′-dG and (5′R) 8,5′-cyclo-2′-dG.

A class of endogenous oxidative DNA lesions that has attracted considerable attention for potential roles in human disease and mutagenesis is 8,5′-cyclopurine-2′-deoxynucleoside (cPu) [2]. The occurrence of cyclopurines in affected tissues may serve as a biomarker for disease or cancer risk, and effectiveness of therapeutic drugs [3], [4]. The cPu DNA lesion arises from hydroxyl radical attack of the H5-atom of the sugar moiety leading to a carbon centered radical that reacts with the C8 position of the purine (guanine (G) or adenine (A)), ultimately creating a very stable glycosidic covalent bond in the cyclization reaction (**Fig. 1**). Structural studies indicate that the presence of the cyclopurine lesion in double-stranded DNA perturbs helix twist and base pair stacking [5], [6]. Consistent with the structural results, cPu lesions are corrected by nucleotide excision repair (NER) [7], [8], which is unusual because the vast majority of oxidative DNA base lesions are repaired by base excision repair (BER) [9]; however, the mechanistic steps involved in recognition and verification of a cPu lesion by the NER machinery are not well understood. Biochemical and cellular studies demonstrate that cPu lesions can interfere with replication [10], inhibit gene expression [7], perturb transcription factor binding to cognate recognition sequences [11], and induce transcriptional mutagenesis [12], leading researchers to investigate their role in disease pathology. Xeroderma pigmentosum (XP) Group C and Cockayne syndrome (CS) Group A patient keratinocytes [13], [14] and tissues of CSB knockout mice [15] contained cPu lesions after exposure to low dose ionizing radiation and tissues from CSB knockout mice. It is hypothesized that cPu lesions are involved in XP neurological disease [16]. Furthermore, the stability of cPu base damage *in vivo* is supported by observations that under controlled environmental conditions, cPu lesions accumulate with age in wild-type mice compared to young mice, and also in congenic progeroid Ercc1−/Δ mice deficient in the XPF-ERCC1 endonuclease implicated in NER [17].

While the effects of cPu lesions on the functions of DNA polymerases [18]–[22], DNA nucleases [19], [23], [24], and RNA polymerase II [7] have been determined, there have been no studies on the impact of cPu damage on DNA unwinding enzymes known as helicases. Helicases represent a prominent class of proteins in cellular nucleic acid metabolism that are important in not only DNA replication and transcription, but also DNA repair, recombination, and chromosome segregation; moreover, a number of genetic disorders characterized by age-related symptoms and cancer are linked to mutations in helicase genes [25]. Covalent or noncovalent DNA modifications that alter helicase function are thought to play a role in processes involving replication stress, DNA damage signaling, and DNA repair [26]. In terms of eukaryotic NER, the XPD helicase is believed to play an instrumental role in DNA damage verification that is necessary for subsequent steps to process and replace the damaged DNA with correct nucleotides [27], [28]. Because helicases are now widely recognized as key enzymes in processes that are either directly affected by DNA damage or are themselves implicated in the DNA damage response, we have carefully examined the potential effects of cPu lesions on the DNA unwinding function of helicases which play a role in human disease. Our findings from biochemical studies with purified recombinant DNA helicases and defined DNA substrates harboring a site- and strand-specific cPu lesion provide the first evidence for their unique and wide ranging effects on DNA helicases that are highly likely to encounter the abundant and stable oxidized base damage.

## Materials and Methods

### Recombinant DNA helicase proteins

Recombinant human FANCJ [29], DDX11 (ChlR1) [30], RECQ1 [31], WRN [32], *E. coli* (Ec) EcDnaB [33], EcDinG [34], and *Thermoplasma acidophilum* (ta) XPD [35] were purified as previously described. EcRecQ was purchased from Abcam. Human recombinant BLM protein was kindly provided by Dr. Ian Hickson (University of Copenhagen). EcUvrD protein was kindly provided by Drs. Ting Xu and Wei Yang (NIDDK, National Institutes of Health). Superfamily designation and polarity for each helicase utilized in this study is provided in **Table 1**.

**Table 1:** DNA Helicases used in this study.

Superfamily	Helicases	Polarity
**1**	EcUvrD	3′ to 5′
**2**	BLM, RECQ1, WRN, EcRecQ	3′ to 5′
	EcDinG, DDX11, FANCJ, taXPD	5′ to 3′
**4**	EcDnaB	5′ to 3′

### DNA substrates

Cyclo dA and Cyclo dG phosphoramidites were purchased from Berry & Associates (Dexter, MI). Synthesis and purification by polyacrylamide gel electrophoresis (PAGE) of oligonucleotides including those which used cyclo dA or cyclo dG phosphoramidites for synthesis was performed by Loftstrand Labs (Rockville, MD). The DNA substrates used were 5′-^32^P-end-labeled partial duplex forked substrates labeled and annealed as described previously [36]. The forked substrates contained 5′ and 3′ single-stranded tails of 41 nucleotide (nt) and a 25 base pair (bp) duplex region. The sequences of the DNA substrates are provided in **Table S1**.

### Standard helicase assays

Helicase reactions were carried out in 20 µl volumes which contained 10 fmol of the forked duplex DNA substrate carrying the cyclopurine lesion either in the top, bottom, or neither strand. The reactions were performed using previously described conditions (FANCJ [37], DDX11 (ChlR1) [30], taXPD [35], [38], RECQ1 [31], WRN [32] EcRecQ [31], BLM [39], EcDnaB [33], EcDinG [34], and EcUvrD [40]). Unless stated otherwise, each of the helicase reactions were carried out by adding the indicated concentration of helicase protein to the reaction mixture followed by incubation at the appropriate temperature for the specified period of time. FANCJ, DDX11, taXPD, RECQ1, WRN, Ec RecQ, EcDnaB, EcDinG and EcUvrD were incubated for 15 min, while BLM was incubated for 30 min. Reactions were quenched with 20 µl of 2X Stop Buffer, containing 17.5 mM EDTA, 0.6% SDS, 0.02% bromophenol blue, 0.02% xylene cyanol and 10-fold excess of unlabeled oligonucleotide which contained the same sequence as the labeled strand. The unlabeled oligonucleotide was added to prevent reannealing. The quenched helicase reaction mixture samples were electrophoresed on non-denaturing 12% polyacrylamide (19∶1 acrylamide-bisacrylamide) gels, visualized using a PhosphorImager, and quantified with ImageQuant Sofware.

### Sequestration helicase assays

For helicase sequestration experiments, FANCJ (9.6 nM) or RECQ1 (8.8 nM) was preincubated for 3 min at 30°C or 37°C, respectively, with ATP (2 mM) and the indicated amounts of unlabeled forked duplex competitor DNA substrates containing the cdA lesion in the top strand, bottom strand, or neither strand. Subsequently, 10 fmol of radiolabeled 19 bp forked duplex, also known as the tracker substrate [41], was added to the mixture and incubated for an additional 10 min at 30°C (FANCJ) or 37°C (RECQ1). The helicase reactions were then quenched and resolved on 12% polyacrylamide gels and visualized as described under “Standard helicase assays”.

### Protein trap kinetic helicase assays

For protein trap kinetic helicase assays, FANCJ (0.6 nM), DDX11 (1 nM), EcUvrD (1 nM), and RECQ1 (7 nM) were preincubated for 3 min at 24°C with 5 nM of the radiolabeled forked duplex DNA substrate carrying the cyclo dA lesion in the top, bottom, or neither strand. After 3 min, ATP (2 mM) and 500 nM oligo dT_200_ (to serve as protein trap) was added simultaneously to the reaction mixture and incubated at 30°C or 37°C. Aliquots (20 µl) of the reaction mixture were quenched at 10-sec intervals with 2X Stop Buffer containing a 10-fold excess of unlabeled oligonucleotide with the same sequence as the labeled strand. Products of helicase reaction mixtures were then resolved on 12% polyacrylamide gels and visualized as described under “Standard helicase assays”.

## Results

Up to this point, there has been no assessment of the effect of cPu base damage on DNA unwinding by any helicase. Given that cPu lesions arising from oxidative stress are believed to be fairly abundant [2], [16], [17], [42], [43], interfere with DNA replication and transcription, and play a prominent role in mutagenesis and human disease, we undertook a systematic investigation of the effect of cPu on DNA unwinding by purified recombinant DNA helicases from different classifications based on sequence homology (**Table 1**). The DNA substrates used for this study are composed of partially complementary single-stranded oligonucleotides containing a single cPu (dA or dG) in either the top or bottom strand within the double-stranded region of a forked duplex. Nine bp reside between the 41-nucleotide single-stranded tails and the site of the cPu adduct and 15 bp reside between the lesion and blunt duplex end on the opposite side of the DNA substrate (**Table S1**). The control substrate consisted of the same oligonucleotides except there was no cPu lesion present in either strand.

### Effects of a cyclopurine lesion on DNA unwinding by RecQ helicases under multi-turnover conditions

**Figure 2:**
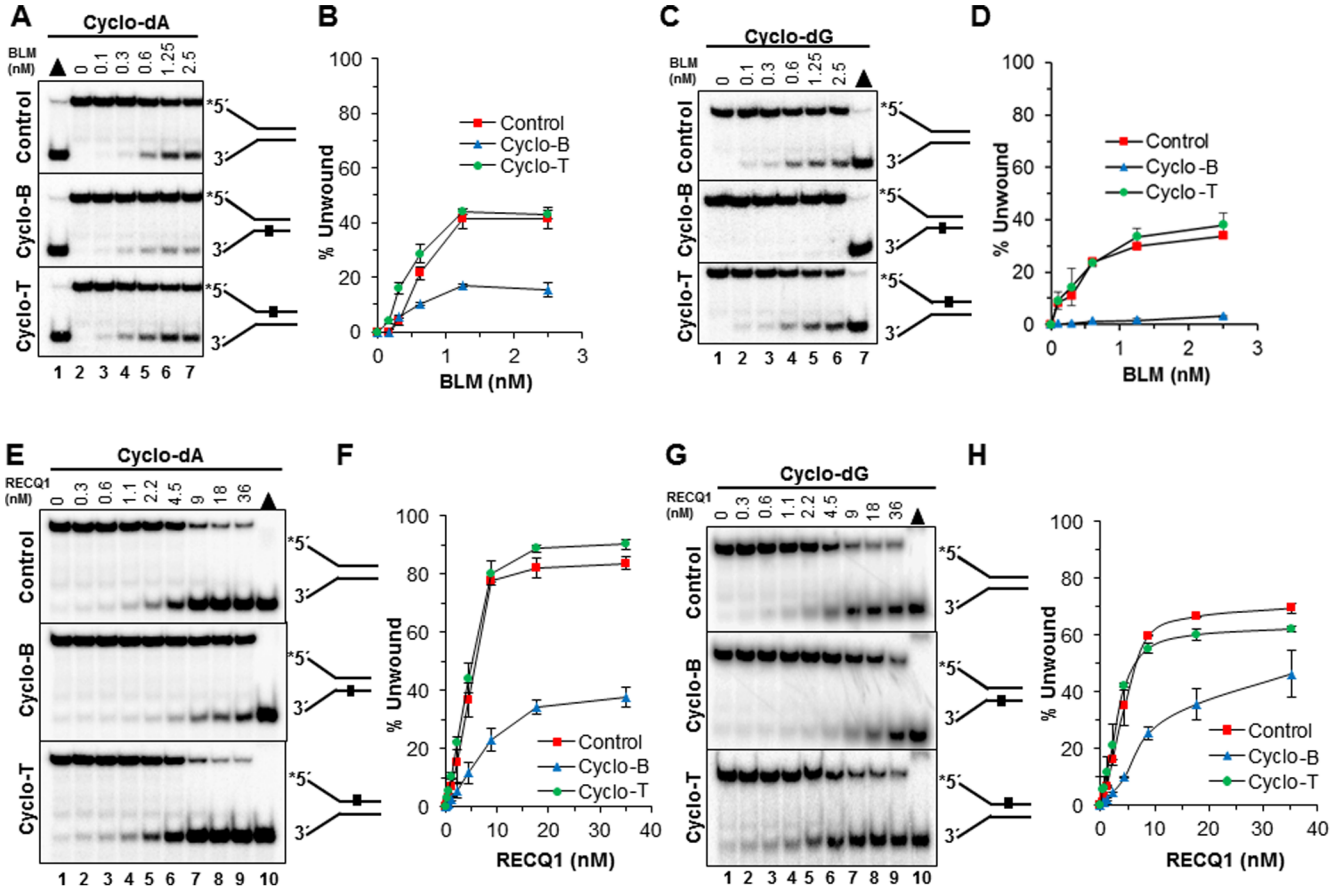
Effect of a site- and strand-specific cyclopurine lesion on BLM or RECQ1 helicase activity. Helicase reactions were carried out by incubating the indicated BLM or RECQ1 concentrations with 0.5 nM forked duplex DNA that contained a cyclopurine lesion in the top strand (nontranslocating-Cyclo T), bottom strand (translocating-Cyclo B), or neither strand (Control) at 37°C for 15 min (RECQ1) or 30 min (BLM) under standard helicase assay conditions described in the Materials and Methods. *A,* BLM unwinding of undamaged and cyclo dA damaged DNA substrates. Lane 1, heat-denatured DNA substrate control; lane 2, no enzyme control; lanes 3–7, indicated concentrations of BLM. *B,* Quantification of BLM helicase activity on cdA substrates with error bars. *C,* BLM unwinding of undamaged and cyclo dG damaged DNA substrates. Lane 1, no enzyme control; lanes 2–6, indicated concentrations of BLM, lane 7 heat-denatured DNA substrate control. *D,* Quantification of BLM helicase activity on cdG substrates with error bars. *E,* RECQ1 unwinding of undamaged and cyclo dA damaged DNA substrates. Lane 1, no enzyme control; lanes 2–9, indicated concentrations of RECQ1; lane 10, heat-denatured DNA substrate control. *F,* Quantification of RECQ1 helicase activity on cdA substrates with error bars. *G,* RECQ1 unwinding of undamaged and cyclo dG damaged DNA substrates. Lane 1, no enzyme control; lanes 2–9, indicated concentrations of RECQ1; lane 10, heat-denatured DNA substrate control. *H,* Quantification of RECQ1 helicase activity on cdG substrates with error bars.

We began with the human RecQ DNA helicase BLM implicated in the hereditary chromosomal instability disorder Bloom Syndrome, and also the human RECQ1 DNA helicase which is not yet reported to be genetically linked to a human disease but thought to play a role in cancer suppression [44]. BLM unwound the control DNA substrate and the substrate with the cyclo dA (**Fig. 2A**) or cyclo dG (**Fig. 2C**) lesion in the top (non-translocating) strand similarly and in a protein concentration dependent manner. The substrate with the cyclo dA (**Fig. 2A, B**) or cyclo dG (**Fig. 2C, D**) lesion in the bottom (translocating) strand was poorly unwound, especially for the cyclo dG adduct in which only 2% substrate was unwound compared to nearly 40% of the control substrate or substrate with the non-translocating strand cyclo dG lesion.

**Figure 3:**
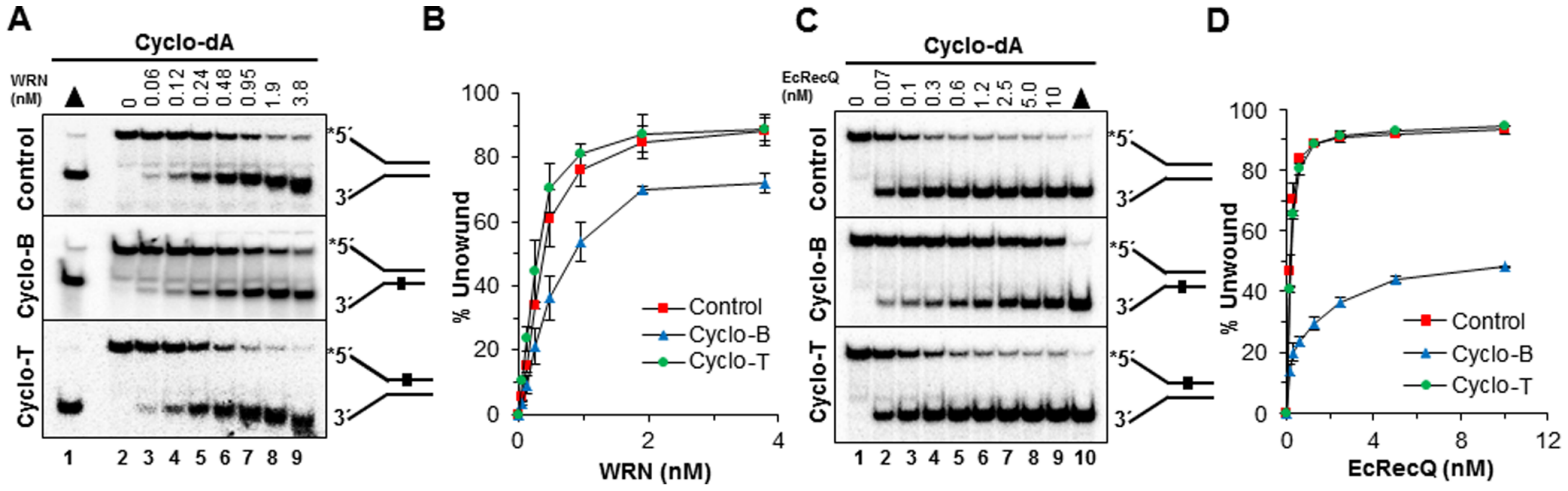
Effect of a site- and strand-specific cyclopurine lesion on WRN or EcRecQ helicase activity. Helicase reactions of 20 µl were carried out by incubating the appropriate WRN or EcRecQ concentrations with 0.5 nM forked duplex DNA that contained a cyclopurine lesion in the top strand (nontranslocating-Cyclo T), bottom strand (translocating-Cyclo B), or neither strand (Control) at 37°C for 15 min under standard helicase assay conditions described in the Materials and Methods. *A,* WRN unwinding of undamaged and cyclo dA damaged DNA substrates. lane 1, heat denatured DNA substrate control, lane 2 no enzyme control, lane 3–9, indicated concentrations of WRN. *B,* Quantification of WRN helicase activity on cdA substrates with error bars. *C,* EcRecQ unwinding of undamaged and cyclo dA damaged DNA substrates. Lane 1, no enzyme control; lanes 2–9, indicated concentrations of EcRecQ; lane 10, heat-denatured DNA substrate control. *D,* Quantification of EcRecQ helicase activity on cdA substrates with error bars.

RECQ1 was inhibited by the cyclo dA in the translocating strand, showing nearly a four-fold reduced level of helicase activity at the 9 nM RECQ1 concentration where 80% of the control substrate as well as the substrate with the lesion in the non-translocating strand was unwound, compared to only 20% of the substrate with the cyclo dA in the translocating strand (**Fig. 2E, F**). RECQ1 was also similarily inhibited by the cyclo dG substrates, although the differences were less dramatic compared to cyclo dA (**Fig. 2G, H**). We also tested another human RecQ helicase, WRN which is implicated in Werner Syndrome [45]. Analysis of reaction products from ATP-dependent WRN helicase assays revealed partial inhibition by the cyclo dA in the translocating strand (**Fig. 3A, B**), but not to the extent as observed for BLM or RECQ1 helicase. EcRecQ helicase was also affected by the cyclo dA in a strand-specific manner, showing inhibition only when the adduct was positioned in the translocating strand (**Fig. 3C, D**). Collectively, the results from DNA unwinding assays with human BLM, WRN, RECQ1, and EcRecQ demonstrated that the RecQ helicases were inhibited by the cPu to different extents when the lesion resided in the helicase translocating strand of the forked duplex DNA molecule.

### A cyclopurine lesion does not inhibit DNA unwinding by Superfamily 1 or 4 DNA helicases under multi-turnover conditions

**Figure 4:**
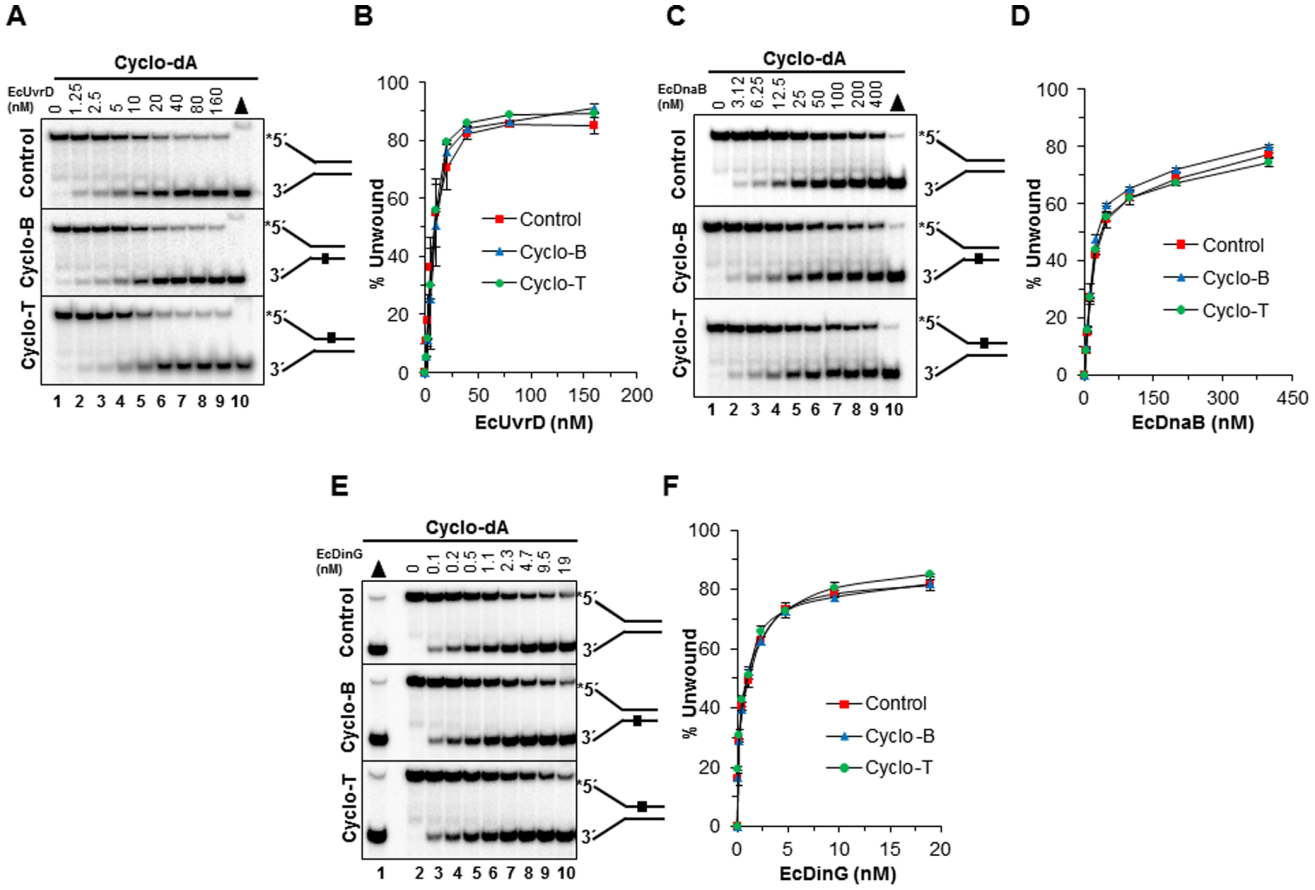
Effect of a site- and strand-specific cyclopurine lesion on EcUvrD, EcDnaB or EcDinG helicase activity. Helicase reactions were carried out by incubating the appropriate EcUvrD, EcDnaB or EcDinG concentrations with 0.5 nM forked duplex DNA that contained a cyclopurine lesion in the top strand (nontranslocating-Cyclo T), bottom strand (translocating-Cyclo B), or neither strand (Control) at 37°C for 15 min under standard helicase assay conditions described in the Materials and Methods. *A,* EcUvrD unwinding of undamaged and cyclo dA damaged DNA substrates. Lane 1, no enzyme control; lanes 2–9, indicated concentrations of EcUvrD; lane 10, heat-denatured DNA substrate control. *B,* Quantification of EcUvrD helicase activity on cdA substrates with error bars. *C,* EcDnaB unwinding of undamaged and cyclo dA damaged DNA substrates. Lane 1, no enzyme control; lanes 2–9, indicated concentrations of EcDnaB; lane 10, heat-denatured DNA substrate control. *D,* Quantification of EcDnaB helicase activity on cdA substrates with error bars. *E,* EcDinG unwinding of undamaged and cyclo dA damaged DNA substrates. Lane 1, heat-denatured DNA substrate control; lane 2, no enzyme control; lanes 3–10, indicated concentrations of EcDinG. *F,* Quantification of EcDinG helicase activity on cdA substrates with error bars.

Our observations that a single cyclopurine residing in the duplex was able to inhibit unwinding by all the RecQ helicases tested led us to ask what the effect of a cyclopurine would be on DNA unwinding by EcUvrD, a SF1 bacterial helicase implicated in NER and mismatch repair [46]. Here it is relevant that the major contacts between SF1 helicases and DNA are believed to be with the bases via hydrophobic interactions in contrast to SF2 helicase in which electrostatic interactions between the ionic side chains of amino acids in the helicase protein and the negatively charged sugar-phosphate backbone prevail [47]. Surprisingly, EcUvrD was resistant to any detectable inhibition by the cyclopurine adduct residing in either the translocating or non-translocating strands of the DNA substrate (**Fig. 4A, B**).

We next tested the SF4 DNA helicase EcDnaB, a hexameric ring-like helicase that is responsible for unwinding complementary strands at the replication fork in *E. coli*. EcDnaB unwinds forked duplex DNA by inserting one strand within the donut hole of the hexamer and extruding the other strand outside the central channel [48]. Therefore, it is believed that the replicative helicase unwinds duplex DNA by a fundamentally different mechanism from a number of DNA repair helicases that operate as monomers or dimers. Experimental studies with EcDnaB and the forked duplex substrates containing the cyclo dA lesion demonstrated that EcDnaB, like the SF1 helicase EcUvrD, was unaffected by the cyclopurine residing in either the translocating or non-translocating strands (**Fig. 4C, D**). Based on these results, we conclude that the sensitivity of SF2 RecQ helicases to a single cyclopurine lesion residing in the duplex is not generally observed by representative DNA helicases from SF1 or SF4.

### Effects of a cyclopurine lesion on DNA unwinding by Fe-S cluster helicases under multi-turnover conditons

**Figure 5:**
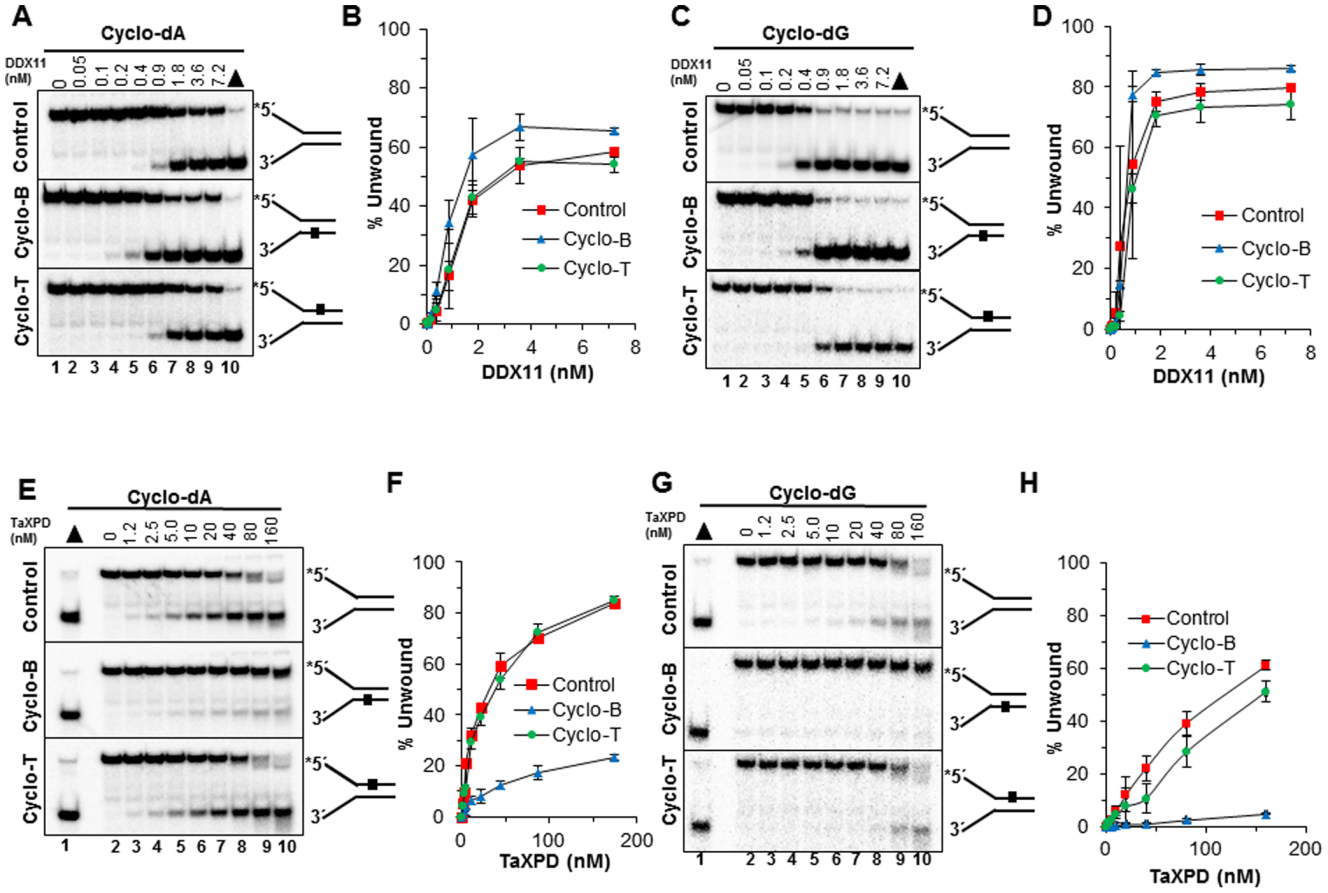
Effect of a site- and strand-specific cyclopurine lesion on DDX11 or taXPD helicase activity. Helicase reactions of 20 µl were carried out by incubating the appropriate DDX11 or taXPD concentrations with 0.5 nM forked duplex DNA that contained a cyclopurine lesion in the top strand (translocating-Cyclo T), bottom strand (nontranslocating-Cyclo B), or neither strand (Control) at 37°C, for 15 min under standard helicase assay conditions described in the Materials and Methods. *A,* DDX11 unwinding of undamaged and cyclo dA damaged DNA substrates. Lane 1, no enzyme control; lanes 2–9, indicated concentrations of DDX11; lane 10, heat-denatured DNA substrate control. *B,* Quantification of DDX11 helicase activity on cdA substrates with error bars. *C,* DDX11 unwinding of undamaged and cyclo dG damaged DNA substrates. Lane 1, no enzyme control; lanes 2–9, indicated concentrations of DnaB; lane 10, heat-denatured DNA substrate control. *D,* Quantification of DDX11 helicase activity on cdG substrates with error bars. *E,* taXPD unwinding of undamaged and cyclo dA damaged DNA substrates. Lane 1, heat-denatured DNA substrate control; lane 2, no enzyme control; lanes 3–10, indicated concentrations of taXPD. *F,* Quantification of taXPD helicase activity on cdA substrates with error bars. *G,* taXPD unwinding of undamaged and cyclo dG damaged DNA substrates. Lane 1, heat-denatured DNA substrate control; lane 2, no enzyme control, lanes 3–10, indicated concentrations of taXPD. *H,* Quantification of taXPD helicase activity on cdG substrates with error bars.

We next tested the *E. coli* Fe-S cluster helicase, DinG. EcDinG was resistant to the cyclo dA lesion in either strand (**Fig. 4E, F**). Also tested were three Fe-S cluster DNA helicases important for chromosomal stability and implicated in genetic diseases: DDX11 (ChlR1) linked to Warsaw Breakage syndrome [49], archaeal *Thermoplasma acidophilum* XPD (taXPD), whose human homologue is linked to Xeroderma Pigmentosum [50], and FANCJ linked to Fanconi Anemia and associated with breast cancer [51]. The control undamaged DNA substrate was unwound by DDX11 in a protein concentration–dependent manner, achieving 60–80% substrate unwound at the highest protein concentrations (**Fig. 5A, C**). The presence of a cyclo dA lesion in either the top or bottom strand did not inhibit DDX11 helicase activity throughout the protein titration. In fact, DDX11 unwinding of the substrate with cyclo dA in the bottom (non-translocating) strand was slightly better than the control substrate or the substrate with the lesion in the top strand (**Fig. 5B**). Similar observations were made for DDX11 helicase activity on the forked duplex substrate series with the cyclo dG lesion (**Fig. 5D**).

The modest effects of a cyclopurine seen with EcDinG and DDX11 led us to investigate how taXPD might behave when it encounters the cycloadduct damage. This question was particularly interesting to us because taXPD is believed to play a critical role in DNA damage verification during a relatively early step of NER [50]. Given the biochemical evidence that cPu is a substrate for the NER pathway [7], [52], we investigated the ability of taXPD to unwind forked duplex substrates with cyclo dA in the top (translocating) or bottom (non-translocating) strands. As shown in **Fig. 5E, F**, taXPD was strongly inhibited by the cyclo dA in the non-translocating strand. Similar behavior with taXPD was also seen with the cyclo dG substrates (**Fig. 5G, D**). At 40 nM taXPD, less than 10% of the forked duplex with cyclo dA in the non-translocating strand was unwound compared to 60% of the control forked duplex or the substrate with cyclo dA in the translocating strand.

**Figure 6:**
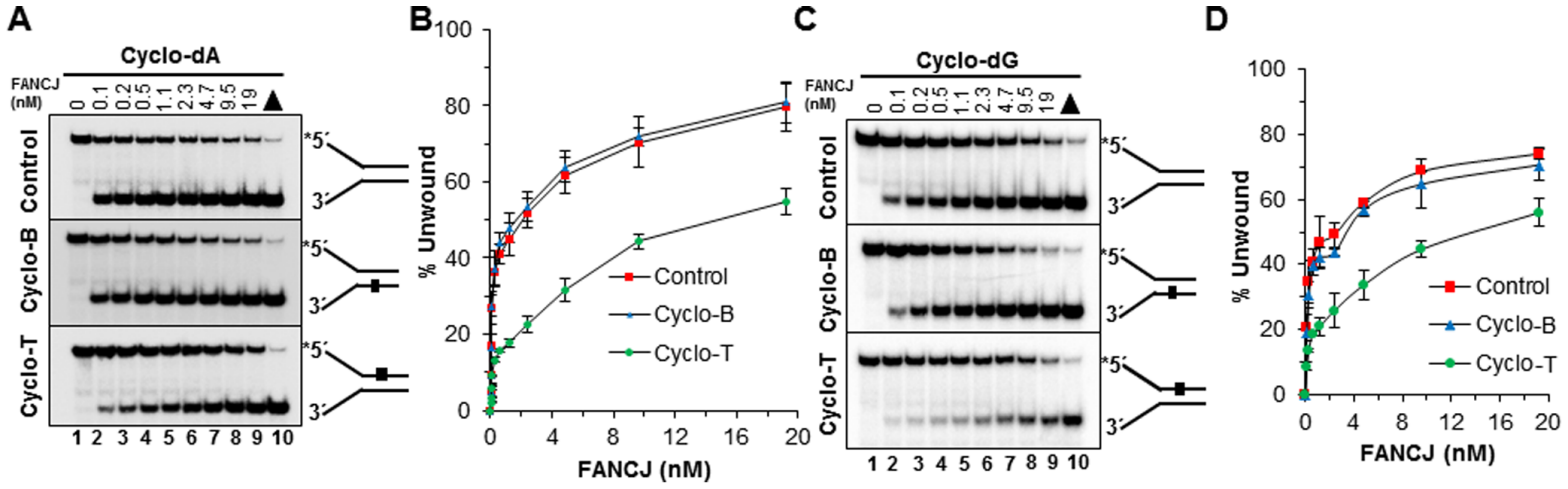
Effect of a site- and strand-specific cyclopurine lesion on FANCJ helicase activity. Helicase reactions were carried out by incubating the appropriate FANCJ concentrations with 0.5 nM forked duplex DNA that contained a cyclopurine lesion in the top strand (translocating-Cyclo T), bottom strand (nontranslocating-Cyclo B), or neither strand (Control) at 30°C for 15 min under standard helicase assay conditions described in the Materials and Methods. *E,* FANCJ unwinding of undamaged and cyclo dA damaged DNA substrates. Lane 1, no enzyme control; lanes 2–9 indicated concentrations of FANCJ; lane 10, heat- denatured DNA substrate control. *F,* Quantification of FANCJ helicase activity on cdA substrates with error bars. *G,* FANCJ unwinding of undamaged and cyclo dG damaged DNA substrates. lane 1, no enzyme control; lanes 2–9, indicated concentrations of FANCJ; lane 10, heat- denatured DNA substrate control. *H,* Quantification of FANCJ helicase activity on cdG substrates with error bars.

A series of experiments were also performed with FANCJ helicase. FANCJ unwound the DNA substrates in a protein concentration-dependent manner in the 15-min incubation. However, in this case there was markedly less unwinding by FANCJ of the DNA substrate harboring the cyclo dA (**Fig. 6A, B**) or cyclo dG (**Fig. 6C, D**) in the top (translocating) strand. Throughout the FANCJ protein titration range of 0.15–2.4 nM, there was consistently a 2- to 3-fold better unwinding of the control undamaged DNA substrate compared to the substrate with either cyclo dA or cyclo dG in the helicase translocating strand. Based on these results, we conclude that the Fe-S cluster helicases are differentially affected by the cyclopurine lesions, with taXPD being impeded by either cyclo dA or cyclo dG in the non-translocating strand, inhibition of FANCJ helicase activity by the translocating strand cyclo dA or cyclo dG lesion, and no effect of the cyclopurine lesion on DDX11 or EcDinG.

### Sequestration studies with SF2 helicases and forked duplex competitor DNA harboring a cyclopurine lesion

**Figure 7:**
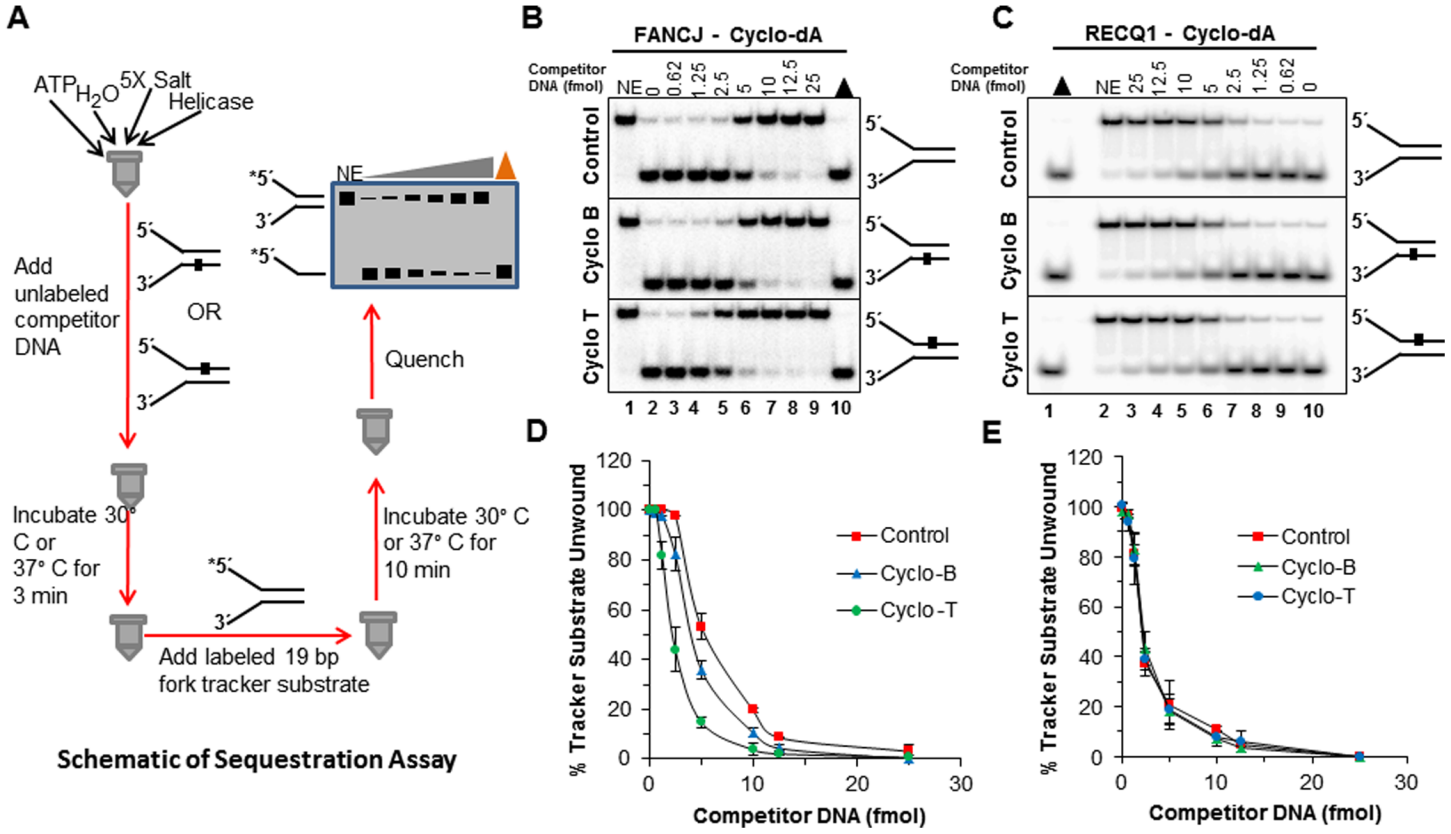
Sequestration of FANCJ, but not RECQ1, by cyclo dA. *A,* Schematic of sequestration assay. Sequestration assays were performed with 9.6 nM FANCJ or 8.8 nM RECQ1 and the indicated concentrations of the competitor DNA forked duplex at 30°C (FANCJ) or 37°C (RECQ1) under sequestration assay conditions described in the Materials and Methods. *B* and *C,* FANCJ (*B*) or RECQ1 (*C*) unwinding of undamaged 19 bp tracker DNA substrate after incubation with unlabeled forked duplex DNA molecules that contained cyclo dA in the top, bottom, or neither strand. *D and E,* Quantification of FANCJ and RECQ1 helicase activity from representative sequestration experiments shown in panels *B* and *C*, respectively.

Previously, we observed that certain forms of DNA damage to the base (*e.g*., thymine glycol) [53] or sugar-phosphate backbone [41] resulted in helicase sequestration by the lesion. To address if this is the case for a cyclopurine, helicase sequestration experiments were performed to evaluate if FANCJ (5′ to 3′ helicase) or RECQ1 (3′ to 5′ helicase) was differentially trapped during unwinding of DNA substrate molecules containing the cyclo dA lesion in the translocating versus non-translocating strand. If the helicase is sequestered when it encounters the cyclo dA in the strand it is predominantly translocating, then the enzyme would be less available to unwind a forked duplex tracker substrate added subsequently to the reaction mixture. A schematic for the experimental procedure is shown in **Fig. 7A**. Throughout the competitor DNA titration range, FANCJ helicase activity on the tracker substrate was inhibited to a significantly greater extent when the helicase was preincubated with the unlabeled forked duplex harboring a cyclo dA lesion in the top (translocating) strand compared to the control undamaged forked substrate (**Fig. 7B, D**). For example, FANCJ was able to only unwind 44% of the tracker substrate when the helicase was preincubated with 2.5 fmol of the forked duplex containing the cyclo dA adduct in the translocating strand whereas 98% of the tracker substrate was unwound when FANCJ was preincubated with the undamaged forked duplex. At this same amount of competitor DNA containing cyclo dA in the non-translocating strand, FANCJ was able to unwind 82% of the tracker substrate. Using 5 fmol of competitor DNA containing the cdA lesion in the translocating strand, only 15% of the tracker substrate was unwound by FANCJ. At this same level of competitor DNA with no damage, FANCJ unwound 53% of the tracker substrate. An intermediate level of FANCJ helicase activity on the tracker substrate was observed when FANCJ was preincubated with the competitor DNA containing the cdA in the non-translocating strand.

In contrast to the experimental results for FANCJ, sequestration experiments performed with RECQ1 demonstrated that the cyclo dA adduct positioned in either the bottom (translocating) or top (non-translocating) strand had no discernible effect on the ability of RECQ1 to unwind the tracker substrate compared to preincubation with the control undamaged forked duplex (**Fig. 7C, E**). Thus, despite the finding that RECQ1 helicase activity is inhibited by the cyclo dA in the translocating strand, RECQ1 is not trapped by the lesion. This would suggest that when RECQ1 becomes blocked by the lesion, it may dissociate from the DNA and become available to bind and unwind the tracker substrate.

### Protein trap kinetic helicase assays with cyclopurine substrate to simulate single-turnover conditions

**Figure 8:**
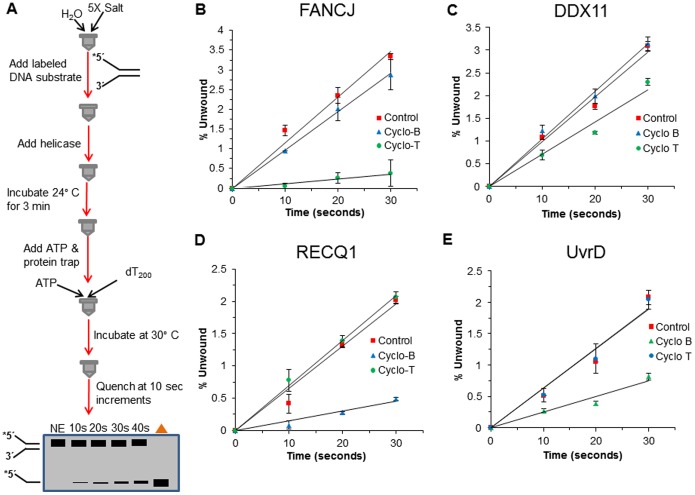
Protein trap kinetics assay to measure FANCJ, DDX11, RECQ1, and EcUvrD rates of unwinding DNA substrates with cyclo dA. Reactions were performed under protein trap kinetic assay kinetics conditions described in the Materials and Methods. *A,* Schematic of protein trap kinetics helicase assay. *B*, Quantification of FANCJ helicase activity on cdA DNA substrates. *C*, Quantification of DDX11 helicase activity on cdA DNA substrates. *D,* Quantification of RECQ1 helicase activity on cdA DNA substrates. *E,* Quantification of EcUvrD helicase activity on cdA DNA substrates.

**Figure 9:**
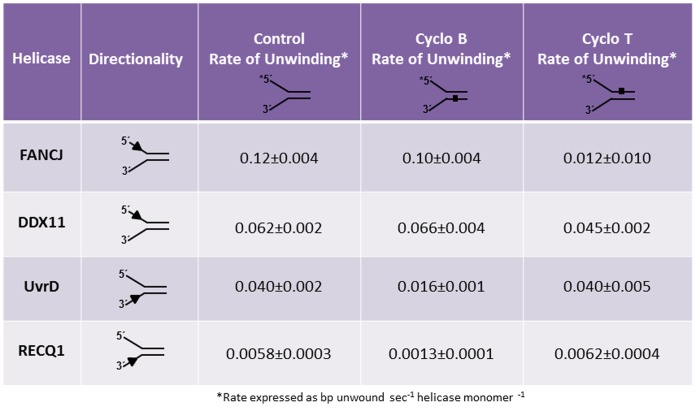
Protein trap kinetic rates for FANCJ, DDX11, RECQ1, and EcUvrD. Rates of unwinding were calculated for each helicase on undamaged DNA substrates and DNA substrates containing a cyclo dA damage in the translocating or non-translocating strand. Rates were determined based on experimental data from Figure 8.

To further reexamine the effect of a cyclopurine lesion on helicase activity, we performed kinetic assays with a protein trap to stimulate single turnover conditions (**Fig. 8A**). FANCJ (0.6 nM), DDX11 (1 nM), RECQ1 (7 nM), or EcUvrD (1 nM) were allowed to bind the 5 nM forked duplex substrate containing the cyclo dA lesion in the top, bottom, or neither strand. Helicase reactions were initiated by the simultaneous addition of ATP and 500 nM oligo dT_200_ which served as a protein trap to bind helicases free in solution, helicases that dissociated from the DNA substrate, or helicases that completed unwinding of the forked duplex and dissociated from the single-stranded unwound products during the reaction incubation phase. Reaction mixture samples were removed at 10-sec intervals to establish initial linear rates of DNA unwinding by FANCJ, DDX11, RECQ1, and EcUvrD (**Fig. 8B, 8C, 8D, 8E**). Under these conditions, we determined that FANCJ unwound the control undamaged forked duplex substrate at a rate of 0.12 bp sec^-1^ FANCJ monomer^-1^ (**Fig. 9**). The forked duplex substrate with cyclo dA in the top, translocating strand was unwound at a rate of 0.012 bp sec^-1^ FANCJ monomer^-1^, a 10-fold slower kinetics compared to the control substrate. In contrast to these results for FANCJ, the rate of DDX11 helicase activity was only reduced 1.4-fold by the translocating strand cyclo dA (**Fig. 9**). The forked duplex with cyclo dA in the bottom, non-translocating strand was unwound relatively efficiently compared to the control substrate for both FANCJ and DDX11. Like the Fe-S cluster helicases, the rates of DNA unwinding under single-turnover conditions for the 3′ to 5′ helicases EcUvrD and RECQ1 were only affected by cyclo dA in the translocating strand, (which in these cases would be the bottom strand) (**Fig. 9**); however, the inhibition by cyclo dA was greater for RECQ1 (4.5-fold) compared to EcUvrD (2.5-fold). Based on these results, we conclude that a single cyclo dA lesion in the helicase translocating strand of the 25-bp forked duplex substrate significantly slowed down FANCJ and RECQ1 unwinding but only modestly affected DDX11 and EcUvrD.

## Discussion

The strong ability of cPu lesions to block replication and transcription, coupled with their accumulation with aging or progeria in mice, has compelled researchers to understand how cPu lesions exert their cytotoxic effects and how efficiently they are detected and corrected by the cellular DNA repair machinery. Their structural perturbation to the DNA double helix is similar to that of the UV-induced photoproduct cyclobutane pyrimidine dimer (CPD) [54], [55], which may help to explain why the oxidatively induced cPu is a substrate of NER [7], [52]. In addition, a recent study suggests that cdA adducts found at 3′ termini of double-strand breaks can be removed by human Apurinic Endonuclease 1 (APE1), suggesting an alternative pathway for cleansing genomic cPu lesions [24]. Due to their distinctive biological effects as well as their impressive chemical stability and distorting effects on the DNA helix, we set out to assess in a systematic manner the effects of cyclo dA or cyclo dG adducts on DNA unwinding catalyzed by a panel of helicases, a number of which are defective in hereditary disorders and are implicated in pathways required to preserve genomic stability. These efforts have led us to conclude that the effects of cyclopurine lesions on helicase-catalyzed DNA unwinding are of quite a broad range, and to some extent not easily classified according to helicase family or even within the same species of conserved helicase proteins. The RecQ helicases (human BLM, RECQ1, WRN, and EcRecQ) behaved in the most predictable manner in which the cPu lesion residing in the translocating strand was found to be inhibitory to DNA unwinding, whereas there was little to no effect of the cPu in the non-translocating strand on helicase function. In contrast, the cPu lesion exerted non-uniform effects on DNA unwinding by Fe-S cluster helicases that varied widely in terms of strand-specificity and damage recognition among those helicases tested (FANCJ, ChlR1 (DDX11), taXPD, and EcDinG). Finally, under multi-turnover conditions DNA unwinding by the classic *E. coli* DNA repair helicase UvrD (which is believed to unwind double-stranded DNA as a homo-dimer [56]) or the replicative hexameric ring-like helicase EcDnaB [57] was not affected in any significant manner by the cPu lesion irrespective of strand residence.

In order to further examine the effect that cPu had on helicase activity, rates of unwinding for select helicases were conducted under single-turnover conditions. The SF2 Fe-S cluster helicase FANCJ is strongly inhibited by the cyclopurine lesion, a result that is consistent with the strand-specific inhibition observed under the multi-turnover conditions. In contrast, the rate of DNA unwinding by the Fe-S cluster helicase DDX11 is only modestly affected by the cyclopurine damage, a result that is also consistent with what was observed under multi-turnover conditions. Thus, DDX11 possesses an intrinsic ability to unwind and bypass the cyclopurine, even in the translocating strand, without invoking the requirement for additional helicase molecules to load on the same substrate and help stalled DDX11 helicase molecule translocate forward. In the case of 3′ to 5′ helicases, SF1 helicase EcUvrD is detectably affected by the translocating strand cyclopurine under single-turnover conditions, whereas the enzyme fully tolerated the lesion under multi-turnover conditions. These results suggest that the ability of EcUvrD to efficiently unwind the DNA substrate with the translocating strand cyclopurine lesion in a protein concentration manner under multi-turnover conditions may be in part due to the loading of more than a single functional helicase molecule on the DNA substrate during the reaction incubation period. In contrast, the 3′ to 5′ helicase RECQ1 is more negatively affected in its ability to unwind the DNA substrate with the translocating strand cyclopurine lesion under either single-turnover or multi-turnover conditions. Thus, inhibition of both RECQ1 and FANCJ stalling by the cyclopurine lesion in the translocating strand is not efficiently overcome by increasing the number of helicase molecules in solution.

The selective deterrence of DNA unwinding by the RecQ helicases when the cPu lesion resided in the translocating strand is interesting in light of recent experimental findings that BLM [58] and Arabidopsis RecQ homologs [59] have the ability to switch strands upon encountering undamaged double-stranded DNA and effectively translocate on the opposite strand away from the duplex. Based on the strand-switching model, it could be suggested that when RecQ helicase molecules encounter a helix-distorting lesion such as cPu, a population of helicase molecules may switch strands and translocate on the opposite strand away from the lesion, enabling the unwound strands to reanneal behind it. Although it is unknown if a DNA lesion causes the acceleration of RecQ strand-switching, it seems probable that strand-specific inhibition of DNA unwinding by a RecQ helicase caused by an adduct such as cPu would increase the probability of the helicase to undergo a strand-switching event. From a biological perspective, cPu lesions are likely to modulate the functional roles of RecQ helicases which are generally believed to involve sensing DNA damage at the replication fork or facilitating the processing of DNA ends or recombinant DNA molecules that arise in early or later steps of double-strand break repair [25].

FANCJ was the sole Fe-S cluster helicase tested that displayed a sensitivity similar to that of the RecQ helicases in which inhibition was observed only when the cPu adduct resided in the helicase translocating strand. To our knowledge, strand-switching by FANCJ or any Fe-S helicase for that matter has not been examined, leaving in question the mechanistic basis for inhibition of FANCJ helicase by the cPu lesion. Unlike most, if not all RecQ helicases, FANCJ does not efficiently catalyze strand annealing of pre-existing complementary single-stranded DNA molecules [37]. If helicase-catalyzed strand annealing is a signature event of strand-switching by a RecQ helicase, FANCJ is likely to behave differently when it encounters helicase roadblocks. Consistent with this notion, FANCJ, but not RECQ1, was partially sequestered by the cPu. Protein trap kinetic assays demonstrated that the rate of FANCJ helicase activity was reduced 10-fold by the translocating strand cyclo dA. In contrast, the sequence-related ChlR1 and EcDinG helicases fully tolerated the cPu lesion in either the translocating or non-translocating strands. Therefore, the Fe-S cluster helicases ChlR1 and EcDinG are likely to unwind damaged DNA by a mechanism that is distinguishable from FANCJ.

Previously, the effects of more classic NER lesions such as the UV photoproduct CPD have been examined for their effects on DNA binding and unwinding by various archaeal XPD helicases [50], [55], [60], [61]. In the current study, we analyzed the effect of a cPu lesion on taXPD helicase activity. taXPD was inhibited by the cPu lesion residing in the helicase non-translocating strand, but unaffected by the cPu in the helicase translocating strand. Recently, it was shown that *Ferroplasma acidarmanus XPD (*faXPD) utilizes its conserved Fe-S cluster domain as a damage sensor pocket, which scans for lesions when translocating on a DNA substrate [50]. Biophysical studies suggest that taXPD utilizes its damage sensor function in order to recognize distinct NER-type lesions at different positions when translocating on a DNA bubble substrate [55]. taXPD preferentially recognized a bulky fluorescein lesion residing in the translocating strand, whereas it detected a CPD lesion located in the non-translocating strand more readily. It was proposed that the fluorescein adduct may distort the helix in a manner that directly impedes passage of single-stranded DNA through the central hole of the helicase protein. In contrast, the CPD lesion may pass through the central hole, but likely comes into contact with the damage sensor when present on the non-translocating strand and thus inhibits taXPD [55]. Based on our results, it is plausible that the cPu lesion is recognized by taXPD in a similar fashion to a CPD lesion.

It is also possible that the differences in sensitivity of Fe-S cluster helicases to DNA damage such as a cPu adduct could be due to other mechanisms. Recent experimental evidence from the Barton lab suggested that DNA repair proteins (*Sulfolobus acidocaldarius* XPD and Endonuclease III) with redox active Fe-S clusters can utilize charge transport along the DNA double helical molecules as a means for detecting DNA lesions and recruiting other repair proteins [62]. It remains to be seen to what extent the cPu lesion affects electron transport along the axis of the DNA double helix, and what role this may play in helicase function or recruitment of other DNA damage repair proteins. Interestingly, the aforementioned APE1 has redox capability which is thought to be important for cell growth and differentiation [63], but it is not well understood if the redox function plays a role in the recognition or excision of oxidized bases.

**Figure 10:**
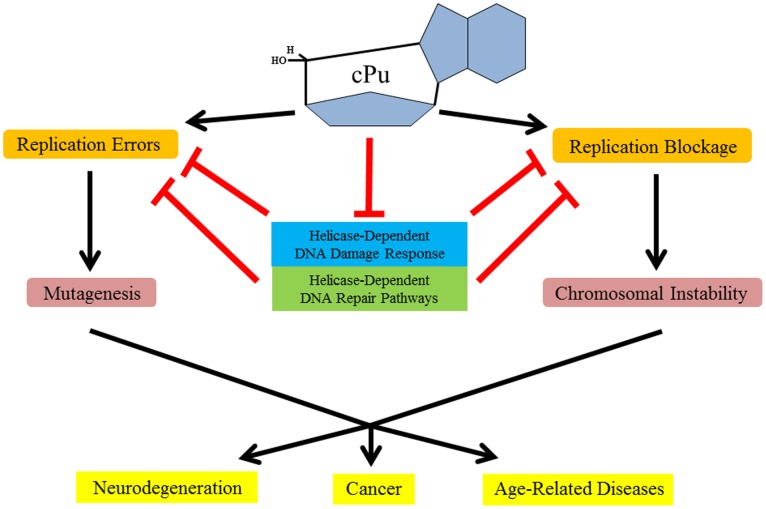
The accumulation of cyclopurine lesions in genomic DNA can cause replication errors or blockage leading to deleterious effects on the fidelity of DNA synthesis and maintenance of genomic stability which have negative outcomes for human health. The functions of certain DNA helicases can be adversely affected by cyclopurines and other forms of DNA damage which potentially impair helicase-dependent DNA damage response and repair pathways. See text for details.

The noted effects of cPu lesions on DNA helicases has implications for how these adducts may have an impact on replication and other areas of DNA metabolism. In a recent study, it was found that cPu adducts inhibit DNA replication and lead to increased mutation frequencies at the lesion sites through base pair transversions [22]. Certain translesion synthesis (TLS) polymerases can promote efficient bypass of the cPu adduct, but can cause mutations at the lesion sites [21], [22]. Interestingly, FANCJ was reported to promote TLS pol eta-dependent bypass of UV-induced DNA damage [64], raising the possibility that the helicase may facilitate TLS past cPu adducts. It is plausible that a helicase like FANCJ which was inhibited by a cPu lesion may help to recruit a TLS polymerase to facilitate DNA synthesis. The ability of cellular DNA replication and repair machinery, including DNA damage response and repair pathways dependent on DNA helicases, to suppress replication errors induced by cyclopurines and other oxidative DNA lesions has important consequences for aging and age-related diseases (**Fig. 10**). It is believed that stem cell function is impaired by age-dependent accumulation of damaged DNA [65]. Extrinsic and intrinsic factors can induce molecular effectors such as reactive oxygen species to cause genomic and epigenomic changes in stem cells that debilitate function, leading to abnormal differentiated cells which in turn contribute to tissue dysfunction and aging [66]. Because cPu adducts have been shown to accumulate in an age-dependent manner [17], it is reasonable to speculate that their metabolism will have important consequences for stem cell dysfunction with aging.

## Supporting Information

Table S1 **DNA substrates used in this study.** Lower case red font “a” or “g” denotes cyclo dA or cyclo dG, respectively.
(DOC)

## References

[1] Berquist BR, Wilson DM III (2012) Pathways for repairing and tolerating the spectrum of oxidative DNA lesions. Cancer Lett 327: 61–72 10.1016/j.canlet.2012.02.001 22353689 PMC3389563

[2] Jaruga P, Dizdaroglu M (2008) 8,5′-Cyclopurine-2′-deoxynucleosides in DNA: mechanisms of formation, measurement, repair and biological effects. DNA Repair (Amst) 7: 1413–1425 10.1016/j.dnarep.2008.06.005 18603018

[3] Anderson KM, Jaruga P, Ramsey CR, Gilman NK, Green VM, et al (2006) Structural alterations in breast stromal and epithelial DNA: the influence of 8,5′-cyclo-2′-deoxyadenosine. Cell Cycle 5: 1240–1244 10.4161/cc.5.11.2816 16760644

[4] Jaruga P, Rozalski R, Jawien A, Migdalski A, Olinski R, et al (2012) DNA damage products (5′R)- and (5′S)-8,5′-cyclo-2′-deoxyadenosines as potential biomarkers in human urine for atherosclerosis. Biochemistry 51: 1822–1824 10.1021/bi201912c 22360777

[5] Huang H, Das RS, Basu AK, Stone MP (2011) Structure of (5′S)-8,5′-cyclo-2′-deoxyguanosine in DNA. J Am Chem Soc 133: 20357–20368 10.1021/ja207407n 22103478 PMC3279155

[6] Zaliznyak T, Lukin M, de los Santos C (2012) Structure and stability of duplex DNA containing (5′S)-5′,8-cyclo-2′-deoxyadenosine: an oxidatively generated lesion repaired by NER. Chem Res Toxicol 25: 2103–2111 10.1021/tx300193k 22928555 PMC3472033

[7] Brooks PJ, Wise DS, Berry DA, Kosmoski JV, Smerdon MJ, et al (2000) The oxidative DNA lesion 8,5′-(S)-cyclo-2′-deoxyadenosine is repaired by the nucleotide excision repair pathway and blocks gene expression in mammalian cells. J Biol Chem 275: 22355–22362 10.1074/jbc.M002259200 10801836

[8] Kuraoka I, Bender C, Romieu A, Cadet J, Wood RD, et al (2000) Removal of oxygen free-radical-induced 5′,8-purine cyclodeoxynucleosides from DNA by the nucleotide excision-repair pathway in human cells. Proc Natl Acad Sci U S A 97: 3832–3837 10.1073/pnas.070471597 10759556 PMC18102

[9] Svilar D, Goellner EM, Almeida KH, Sobol RW (2011) Base excision repair and lesion-dependent subpathways for repair of oxidative DNA damage. Antioxid Redox Signal 14: 2491–2507 10.1089/ars.2010.3466 20649466 PMC3096496

[10] Jasti VP, Das RS, Hilton BA, Weerasooriya S, Zou Y, et al (2011) (5′S)-8,5′-cyclo-2′-deoxyguanosine is a strong block to replication, a potent pol V-dependent mutagenic lesion, and is inefficiently repaired in Escherichia coli. Biochemistry 50: 3862–3865 10.1021/bi2004944 21491964 PMC3092667

[11] Marietta C, Gulam H, Brooks PJ (2002) A single 8,5′-cyclo-2′-deoxyadenosine lesion in a TATA box prevents binding of the TATA binding protein and strongly reduces transcription in vivo. DNA Repair (Amst) 1: 967–975 10.1015/S1568-7864(02)00148-9 12531024

[12] Marietta C, Brooks PJ (2007) Transcriptional bypass of bulky DNA lesions causes new mutant RNA transcripts in human cells. EMBO Rep 8: 388–393 10.1038/sj.embor.7400932 17363972 PMC1852755

[13] D’Errico M, Parlanti E, Teson M, de Jesus BM, Degan P, et al (2006) New functions of XPC in the protection of human skin cells from oxidative damage. EMBO J 25: 4305–4315 10.1038/sj.emboj.7601277 16957781 PMC1570445

[14] D’Errico M, Parlanti E, Teson M, Degan P, Lemma T, et al (2007) The role of CSA in the response to oxidative DNA damage in human cells. Oncogene 26: 4336–4343 10.1038/sj.onc.1210232 17297471

[15] Kirkali G, de Souza-Pinto NC, Jaruga P, Bohr VA, Dizdaroglu M (2009) Accumulation of (5′S)-8,5′-cyclo-2′-deoxyadenosine in organs of Cockayne syndrome complementation group B gene knockout mice. DNA Repair (Amst) 8: 274–278 10.1016/j.dnarep.2008.09.009 18992371 PMC2693312

[16] Brooks PJ (2008) The 8,5′-cyclopurine-2′-deoxynucleosides: candidate neurodegenerative DNA lesions in xeroderma pigmentosum, and unique probes of transcription and nucleotide excision repair. DNA Repair (Amst) 7: 1168–1179 10.1016/j.dnarep.2008.03.016 18495558 PMC2797313

[17] Wang J, Clauson CL, Robbins PD, Niedernhofer LJ, Wang Y (2012) The oxidative DNA lesions 8,5′-cyclopurines accumulate with aging in a tissue-specific manner. Aging Cell 11: 714–716 10.1111/j.1474-9726.2012.00828.x 22530741 PMC3399950

[18] Kamakura N, Yamamoto J, Brooks PJ, Iwai S, Kuraoka I (2012) Effects of 5′,8-cyclodeoxyadenosine triphosphates on DNA synthesis. Chem Res Toxicol 25: 2718–2724 10.1021/tx300351p 23146066

[19] Kuraoka I, Robins P, Masutani C, Hanaoka F, Gasparutto D, et al (2001) Oxygen free radical damage to DNA. Translesion synthesis by human DNA polymerase eta and resistance to exonuclease action at cyclopurine deoxynucleoside residues. J Biol Chem 276: 49283–49288 10.1074/jbc.M107779200 11677235

[20] Pednekar V, Weerasooriya S, Jasti VP, Basu AK (2014) Mutagenicity and Genotoxicity of (5′S)-8,5′-Cyclo-2′-deoxyadenosine in Escherichia coli and replication of (5′S)-8,5′-cyclopurine-2′-deoxynucleosides in vitro by DNA Polymerase IV, Exo-Free Klenow Fragment, and Dpo4. Chem Res Toxicol 27: 200–10 10.1021/tx4002786 24392701 PMC3952113

[21] Swanson AL, Wang J, Wang Y (2012) Accurate and efficient bypass of 8,5′-cyclopurine-2′-deoxynucleosides by human and yeast DNA polymerase eta. Chem Res Toxicol 25: 1682–1691 10.1021/tx3001576 22768970 PMC3423583

[22] You C, Swanson AL, Dai X, Yuan B, Wang J, et al (2013) Translesion synthesis of 8,5′-cyclopurine-2′-deoxynucleosides by DNA polymerases eta, iota, and zeta. J Biol Chem 288: 28548–28556 10.1074/jbc.M113.480459 23965998 PMC3789955

[23] Jaruga P, Theruvathu J, Dizdaroglu M, Brooks PJ (2004) Complete release of (5′S)-8,5′-cyclo-2′-deoxyadenosine from dinucleotides, oligodeoxynucleotides and DNA, and direct comparison of its levels in cellular DNA with other oxidatively induced DNA lesions. Nucleic Acids Res 32: e87 10.1093/nar/gnh087 15215337 PMC443555

[24] Mazouzi A, Vigouroux A, Aikeshev B, Brooks PJ, Saparbaev MK, et al (2013) Insight into mechanisms of 3′-5′ exonuclease activity and removal of bulky 8,5′-cyclopurine adducts by apurinic/apyrimidinic endonucleases. Proc Natl Acad Sci U S A 110: E3071–E3080 10.1073/pnas.1305281110 23898172 PMC3746914

[25] Brosh RM Jr (2013) DNA helicases involved in DNA repair and their roles in cancer. Nature Reviews Cancer 13: 542–58 10.1038/nrc3560 23842644 PMC4538698

[26] Suhasini AN, Brosh RM Jr (2010) Mechanistic and biological aspects of helicase action on damaged DNA. Cell Cycle 9: 2317–2329 10.4161/cc.9.12.11902 20574162 PMC3032018

[27] Egly JM, Coin F (2011) A history of TFIIH: Two decades of molecular biology on a pivotal transcription/repair factor. DNA Repair (Amst) 10: 714–21 10.1016/j.dnarep.2011.04.021 21592869

[28] Fuss JO, Tainer JA (2011) XPB and XPD helicases in TFIIH orchestrate DNA duplex opening and damage verification to coordinate repair with transcription and cell cycle via CAK kinase. DNA Repair (Amst) 10: 697–713 10.1016/j.dnarep.2011.04.028 21571596 PMC3234290

[29] Cantor S, Drapkin R, Zhang F, Lin Y, Han J, et al (2004) The BRCA1-associated protein BACH1 is a DNA helicase targeted by clinically relevant inactivating mutations. Proc Natl Acad Sci U S A 101: 2357–2362 10.1073/pnas.0308717101 14983014 PMC356955

[30] Wu Y, Sommers JA, Khan I, De Winter JP, Brosh RM Jr (2012) Biochemical characterization of Warsaw breakage syndrome helicase. J Biol Chem 287: 1007–1021 10.1074/jbc.M111.276022 22102414 PMC3256869

[31] Sharma S, Sommers JA, Choudhary S, Faulkner JK, Cui S, et al (2005) Biochemical analysis of the DNA unwinding and strand annealing activities catalyzed by human RECQ1. J Biol Chem 280: 28072–84 10.1074/jbc.M500264200 15899892

[32] Sharma S, Otterlei M, Sommers JA, Driscoll HC, Dianov GL, et al (2004) WRN helicase and FEN-1 form a complex upon replication arrest and together process branch-migrating DNA structures associated with the replication fork. Mol Biol Cell 15: 734–750 10.10.91/mbc.E03-08-0567 14657243 PMC329389

[33] Kaplan DL, O’Donnell M (2002) DnaB drives DNA branch migration and dislodges proteins while encircling two DNA strands. Mol Cell 10: 647–657 10.1016/S1097-2765(02)00642-1 12408831

[34] Bharti SK, Sommers JA, George F, Kuper J, Hamon F, et al (2013) Specialization among iron-sulfur cluster helicases to resolve G-Quadruplex DNA structures that threaten genomic stability. J Biol Chem 288: 28217–29 10.1074/jbc.M113.496463 23935105 PMC3784731

[35] Wolski SC, Kuper J, Hanzelmann P, Truglio JJ, Croteau DL, et al (2008) Crystal structure of the FeS cluster-containing nucleotide excision repair helicase XPD. PLoS Biol 6: e149 10.1371/journal.pbio.0060149 18578568 PMC2435149

[36] Brosh RM, Waheed J, Sommers JA (2002) Biochemical characterization of the DNA substrate specificity of Werner syndrome helicase. J Biol Chem 277: 23236–45 10.1074/jbc.M111446200 11956187

[37] Gupta R, Sharma S, Sommers JA, Jin Z, Cantor SB, et al (2005) Analysis of the DNA substrate specificity of the human BACH1 helicase associated with breast cancer. J Biol Chem 280: 25450–25460 10.1074/jbc.M501995200 15878853

[38] Rudolf J, Makrantoni V, Ingledew WJ, Stark MJ, White MF (2006) The DNA Repair Helicases XPD and FancJ have essential iron-sulfur domains. Mol Cell 23: 801–808 10.1016/j.molcel.2006.07.019 16973432

[39] Suhasini AN, Rawtani NA, Wu Y, Sommers JA, Sharma S, et al (2011) Interaction between the helicases genetically linked to Fanconi anemia group J and Bloom’s syndrome. EMBO J 30: 692–705 10.1038/emboj.2010.362 21240188 PMC3041957

[40] Cadman CJ, Matson SW, McGlynn P (2006) Unwinding of forked DNA structures by UvrD. J Mol Biol 362: 18–25 10.1016/j.jmb.2006.06.032 16890954

[41] Suhasini AN, Sommers JA, Yu S, Wu Y, Xu T, et al (2012) DNA repair and replication fork helicases are differentially affected by alkyl phosphotriester lesion. J Biol Chem 287: 19188–19198 10.1074/jbc.M112.352757 22500020 PMC3365951

[42] Mitra D, Luo X, Morgan A, Wang J, Hoang MP, et al (2012) An ultraviolet-radiation-independent pathway to melanoma carcinogenesis in the red hair/fair skin background. Nature 491: 449–453 10.1038/nature11624 23123854 PMC3521494

[43] Wang J, Yuan B, Guerrero C, Bahde R, Gupta S, et al (2011) Quantification of oxidative DNA lesions in tissues of Long-Evans Cinnamon rats by capillary high-performance liquid chromatography-tandem mass spectrometry coupled with stable isotope-dilution method. Anal Chem 83: 2201–2209 10.1021/ac103099s 21323344 PMC3056914

[44] Sharma S, Doherty KM, Brosh RM Jr (2006) Mechanisms of RecQ helicases in pathways of DNA metabolism and maintenance of genomic stability. Biochem J 398: 319–337 10.1042/BJ0060450 16925525 PMC1559444

[45] Monnat RJ Jr (2010) Human RECQ helicases: roles in DNA metabolism, mutagenesis and cancer biology. Semin Cancer Biol 20: 329–339 10.1016/j.semcancer.2010.10.002 20934517 PMC3040982

[46] Kuper J, Kisker C (2013) DNA helicases in NER, BER, and MMR. Adv Exp Med Biol 767: 203–224 10.1007/978-1-4614-5037-510 23161013

[47] Singleton MR, Dillingham MS, Wigley DB (2007) Structure and mechanism of helicases and nucleic acid translocases. Annu Rev Biochem 76: 23–50 10.1146/annurev.biochem.76.052305.115300 17506634

[48] Kaplan DL (2000) The 3′-tail of a forked-duplex sterically determines whether one or two DNA strands pass through the central channel of a replication-fork helicase. J Mol Biol 301: 285–299 10.1006/jmbi.2000.3965 10926510

[49] Bharti SK, Khan I, Banerjee T, Sommers JA, Wu Y, et al (2014) Molecular functions and cellular roles of the ChlR1 (DDX11) helicase defective in the rare cohesinopathy Warsaw breakage syndrome. Cell Mol Life Sci 71: 2625–39 10.1007/s00018-014-1569-4 24487782 PMC4537069

[50] Mathieu N, Kaczmarek N, Ruthemann P, Luch A, Naegeli H (2013) DNA quality control by a lesion sensor pocket of the Xeroderma pigmentosum group D helicase subunit of TFIIH. Current Biology 23: 204–12 10.1016/j.cub.2012.12.032 23352696

[51] Cantor SB, Guillemette S (2011) Hereditary breast cancer and the BRCA1-associated FANCJ/BACH1/BRIP1. Future Oncol 7: 253–261 10.2217/fon.10.191 21345144 PMC3109611

[52] Menoni H, Hoeijmakers JH, Vermeulen W (2012) Nucleotide excision repair-initiating proteins bind to oxidative DNA lesions in vivo. J Cell Biol 199: 1037–1046 10.1083/jcb.201205149 23253478 PMC3529521

[53] Suhasini AN, Sommers JA, Mason AC, Voloshin ON, Camerini-Otero RD, et al (2009) FANCJ helicase uniquely senses oxidative base damage in either strand of duplex DNA and is stimulated by Replication Protein A to unwind the damaged DNA substrate in a strand-specific manner. J Biol Chem 284: 18458–18470 10.1074/jbc.M109.012229 19419957 PMC2709400

[54] Brooks PJ (2007) The case for 8,5′-cyclopurine-2′-deoxynucleosides as endogenous DNA lesions that cause neurodegeneration in xeroderma pigmentosum. Neuroscience 145: 1407–1417 10.1016/j.neuroscience.2006.10.025 17184928 PMC2430073

[55] Buechner CN, Heil K, Michels G, Carell T, Kisker C, et al (2014) Strand-specific recognition of DNA Damages by XPD provides insights into nucleotide excision repair substrate versatility. J Biol Chem 289: 3613–3624 10.1074/jbc.M113.523001 24338567 PMC3916561

[56] Maluf NK, Fischer CJ, Lohman TM (2003) A dimer of Escherichia coli UvrD is the active form of the helicase in vitro. J Mol Biol 325: 913–935 10.1016/S0022-2836(02)01277-9 12527299

[57] Patel SS, Picha KM (2000) Structure and function of hexameric helicases. Annu Rev Biochem 2000 69: 651–97 10.1146/annurev.biochem.69.1.651 10966472

[58] Yodh JG, Stevens BC, Kanagaraj R, Janscak P, Ha T (2009) BLM helicase measures DNA unwound before switching strands and hRPA promotes unwinding reinitiation. EMBO J 28: 405–416 10.1038/emboj.2008.298 19165145 PMC2646154

[59] Klaue D, Kobbe D, Kemmerich F, Kozikowska A, Puchta H, et al (2013) Fork sensing and strand switching control antagonistic activities of RecQ helicases. Nat Commun 4: 2024 10.1038/ncomms3024 23771268 PMC3709500

[60] Mathieu N, Kaczmarek N, Naegeli H (2010) Strand- and site-specific DNA lesion demarcation by the xeroderma pigmentosum group D helicase. Proc Natl Acad Sci U S A 107: 17545–17550 10.1073/pnas.1004339107 20876134 PMC2955138

[61] Rudolf J, Rouillon C, Schwarz-Linek U, White MF (2009) The helicase XPD unwinds bubble structures and is not stalled by DNA lesions removed by the nucleotide excision repair pathway. Nucleic Acids Res 38: 931–41 10.1093/nar/gkp1058 19933257 PMC2817471

[62] Sontz PA, Mui TP, Fuss JO, Tainer JA, Barton JK (2012) DNA charge transport as a first step in coordinating the detection of lesions by repair proteins. Proc Natl Acad Sci U S A 109: 1856–1861 10.1073/pnas.1120063109 22308447 PMC3277573

[63] Li M, Wilson DM III (2014) Human apurinic/apyrimidinic endonuclease 1. Antioxid Redox Signal 20: 678–707 10.1089/ars.2013.5492 23834463 PMC3901322

[64] Xie J, Litman R, Wang S, Peng M, Guillemette S, et al (2010) Targeting the FANCJ-BRCA1 interaction promotes a switch from recombination to poleta-dependent bypass. Oncogene 29: 2499–2508 10.1038/onc.2010.18 20173781 PMC2909592

[65] Behrens A, van Deursen JM, Rudolph KL, Schumacher B (2014) Impact of genomic damage and ageing on stem cell function. Nat Cell Biol 16: 201–207 10.1038/ncb2928 24576896 PMC4214082

[66] Liu L, Rando TA (2011) Manifestations and mechanisms of stem cell aging. J Cell Biol 193: 257–266 10.1083/jcb.201010131 21502357 PMC3080271

