# Supplementary figures and images for: Impact of Age-Associated Cyclopurine Lesions on DNA Repair Helicases
Source: PLoS One. 2014 Nov 19;9(11):e113293. doi: 10.1371/journal.pone.0113293 (PMC4237422; doi:10.1371/journal.pone.0113293)

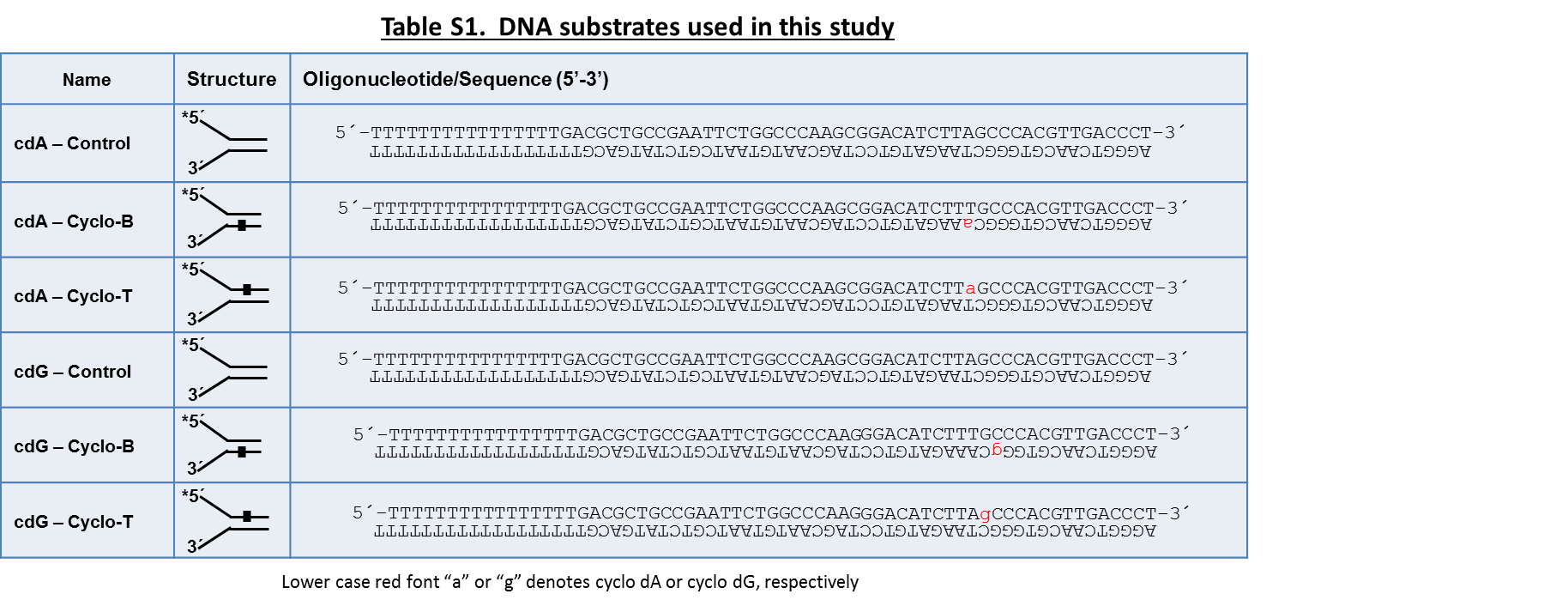

Supplement: Table S1 — DNA substrates used in this study. Lower case red font “a” or “g” denotes cyclo dA or cyclo dG, respectively. (DOC) [file pone.0113293.s001.doc]
